# The syndemic effects of mental ill health, household hunger, and intimate partner violence on adherence to antiretroviral therapy among pregnant women living with HIV in Yaoundé, Cameroon

**DOI:** 10.1371/journal.pone.0246467

**Published:** 2021-02-19

**Authors:** Evette Cordoba, Angela M. Parcesepe, John A. Gallis, Jennifer Headley, Claudian Soffo, Berenger Tchatchou, John Hembling, Joy Noel Baumgartner

**Affiliations:** 1 Department of Epidemiology, Gillings School of Global Public Health, University of North Carolina at Chapel Hill, Chapel Hill, North Carolina, United States of America; 2 School of Nursing, Columbia University, New York, New York, United States of America; 3 Department of Maternal and Child Health, Gillings School of Global Public Health, University of North Carolina at Chapel Hill, Chapel Hill, North Carolina, United States of America; 4 Carolina Population Center, University of North Carolina at Chapel Hill, Chapel Hill, North Carolina, United States of America; 5 Duke Global Health Institute, Duke University, Durham, North Carolina, United States of America; 6 Department of Biostatistics and Bioinformatics, Duke University, Durham, North Carolina, United States of America; 7 Consultant, Yaoundé, Cameroon; 8 Catholic Relief Services, N’Djamena, Chad; 9 Catholic Relief Services, Baltimore, Maryland, United States of America; Christiana Care/University of Delaware, UNITED STATES

## Abstract

**Background:**

This research advances understanding of interrelationships among three barriers to adherence to antiretroviral therapy (ART) among pregnant women living with HIV (WLWH) in Cameroon: probable common mental disorders (CMD), intimate partner violence (IPV), and hunger.

**Methods:**

The sample included 220 pregnant WLWH in Cameroon. Multivariable modified Poisson regression was conducted to assess the relationship between IPV, hunger, and CMD on ART adherence.

**Results:**

Almost half (44%) of participants recently missed/mistimed an ART dose. Probable CMD was associated with greater risk of missed/mistimed ART dose (aRR 1.5 [95% CI 1.1, 1.9]). Hunger was associated with greater risk of missed/mistimed ART dose among those who reported IPV (aRR 1.9 [95% CI 1.2, 2.8]), but not among those who did not (aRR 0.8 [95% CI 0.2, 2.3]).

**Conclusion:**

Suboptimal ART adherence, CMD, and IPV were common among pregnant WLWH in Cameroon. Pregnant WLWH experiencing IPV and hunger may be especially vulnerable to suboptimal ART adherence.

## Introduction

The World Health Organization (WHO) recommends lifelong ART treatment for all pregnant women living with HIV (WLWH), a strategy known as Option B+ [1, 2]. The successful implementation and scale-up of Option B+ has the potential to improve the health of pregnant WLWH and eliminate mother-to-child transmission of HIV. However, suboptimal ART adherence among pregnant WLWH persists throughout sub-Saharan Africa [3, 4]. Since the implementation of Option B+, it has been estimated that one in four pregnant women are lost to follow up within six months of ART initiation [5]. Women who initiate ART during pregnancy are significantly more likely to disengage from HIV care compared to non-pregnant women who initiate ART [6].

In Cameroon, the prevalence of HIV is higher among women compared to men, with women comprising two-thirds of adults living with HIV in Cameroon [7]. Option B+ has been implemented in Cameroon since 2012 [2]. While most pregnant WLWH in Cameroon have access to ART, suboptimal ART adherence remains high [7, 8]. Major barriers to optimal ART adherence persist among pregnant WLWH in sub-Saharan Africa [9, 10].

Mental health disorders are among the most common comorbidities among people living with HIV (PLWH) globally [11, 12]. A systematic review of studies of perinatal depression among African WLWH estimated a mean prevalence of antenatal depression of 23% [13]. Mental health disorders have been consistently associated with suboptimal HIV treatment outcomes among pregnant WLWH, including suboptimal ART adherence [14, 15].

Intimate partner violence (IPV) has also been commonly reported among WLWH and has been associated with suboptimal HIV treatment outcomes, including poor ART adherence, as well as poor mental health [16–21]. Similarly, household hunger and food insecurity have been identified as barriers to ART adherence among pregnant WLWH in sub-Saharan Africa [22, 23]. Food insecurity and household hunger are particularly concerning for pregnant women as pregnant women require increased nutrients to maintain their health, adequately gain weight, and reduce the risk of obstetric and neonatal complications [24, 25].

Although mental health disorders, IPV, and hunger have been independently associated with ART adherence, the interrelationships among these three risk factors remain poorly understood, particularly among pregnant WLWH in sub-Saharan Africa. More specifically, little is known about the intersecting effects of mental health, IPV, and hunger on ART adherence among pregnant WLWH, despite evidence to suggest that meaningful, bidirectional relationships exist among these factors [26]. Syndemic theory posits that certain diseases or morbidities co-occur and synergistically interact, contributing to worse health outcomes among a population [27, 28]. The intersection of substance use, violence, and HIV, known as the SAVA syndemic, was the first syndemic proposed in the literature and has been defined as the “concurrent, intertwined, and mutually reinforcing health and social problems of substance use, violence, and HIV/AIDS [27].” Over the past several decades, evidence to support the existence of the SAVA syndemic has been well-documented [27–29]. In recent years, syndemic frameworks and theory have expanded beyond the SAVA syndemic and have been used to investigate a variety of potentially intersecting health and social problems [29]. The current study applies a syndemic framework to advance understanding the interconnectedness among mental health disorders, IPV, and hunger. Although the original SAVA syndemic focused on substance use, violence, and HIV, the current analysis focuses on violence, mental health, hunger, and HIV. This focus is informed by evidence that metal health, violence, and hunger are commonly experienced, often co-occurring, and consistently associated with ART adherence among pregnant WLWH in resource-limited settings. Greater understanding of the extent to which IPV, CMD and hunger constitute a syndemic among this population of pregnant WLWH can help identify pregnant women particularly vulnerable to suboptimal ART adherence and inform the development and adaptation of integrated strategies to improve ART adherence among this population [26, 30]. This work aims to advance a syndemic understanding of the interrelationships among three known barriers to ART adherence and to investigate if 1) probable common mental disorders (CMD), IPV, or household hunger were independently associated with ART adherence, 2) IPV or household hunger modified the association between probable CMD and ART adherence, and 3) IPV modified the association between household hunger and ART adherence among pregnant WLWH in Yaoundé Cameroon.

## Methods

A cross-sectional analysis was conducted using baseline data collected from a cluster-randomized controlled trial, ‘Evaluating the Impact of Positive Parenting & Early Stimulation within an Integrated Early Childhood Development Program on the Attainment of Developmental Milestones and Health Services Use among HIV-exposed Children in Cameroon’ [ClinicalTrials # NCT03195036]. The sample consisted of 230 pregnant WLWH in Yaoundé, Cameroon in 2017. Eligible participants were women ≥18 years, 7–9 months pregnant at the time of enrollment, willing to be followed up for 21 months, living with HIV, resided in Nkoldongo or Djoungolo districts, and received antenatal care and prevention of mother-to-child transmission (PMTCT) services at one of the 10 selected study clinics. Participants were recruited into the parent study and completed baseline assessments between April and September 2017. The current analysis used baseline data from 220 pregnant WLWH enrolled in the parent study and for whom ART adherence data were available. Data collection consisted of a structured interview during the participants’ third trimester of pregnancy that included questions on mental health, IPV, hunger, and ART adherence. All participants provided written informed consent. The study was approved by the Institutional Review Boards (IRB) of Duke University and the National Ethical Committee of Research for Human Health of Cameroon.

### Mental health

Mental health was measured with the WHO Self-Reporting Questionnaire (SRQ-20). The SRQ-20 is a mental health screening tool which includes questions related to depression, anxiety, and somatic symptoms in the last 30 days [31]. Scores range from 0 to 20 and a cutoff score determines probable common mental disorder. Informed by previous research using the SRQ-20 in sub-Saharan Africa, a score of seven or below was coded as no probable common mental disorder and a score of eight or greater was coded as probable common mental disorder [32, 33].

### Intimate partner violence

Assessment of IPV was based on the Demographic and Health Survey (DHS) violence module used in the 2011 Cameroon DHS and included questions of sexual and physical IPV [34]. Assessment of sexual IPV consisted of two questions about whether the participant’s spouse or partner: *physically forced you to have sexual intercourse with him even when you did not want to or forced you to perform other sexual acts you did not want to in the past 12 months*. Participants who responded yes to either of these questions were categorized as having experienced sexual IPV. Assessment of physical IPV consisted of eight questions about whether the participant’s spouse or partner: *pushed you*, *shaken you*, *or thrown something at you; slapped you; twisted your arm or pulled your hair; punched you with his fist or with something that could hurt you; kicked or dragged you; tried to strangle or burn you; threatened you with a knife*, *gun*, *or other type of weapon; or attacked you with a knife*, *gun*, *or other type of weapon in the past 12 months*. Participants who responded yes to one or more of these questions were categorized as having experienced physical IPV.

### Household hunger

Household hunger was assessed using the Household Hunger Scale [35]. This scale consisted of three questions about household hunger in the past 30 days: *was there ever no food to eat of any kind in your house because of lack of resources to get food; did you or any household member go to sleep at night hungry because there was not enough food; did you or any household member go a whole day and night without eating anything because there was not enough food*. If participants responded affirmatively to any question, they were asked about frequency of the occurrence. This instrument was developed and validated for cross-cultural use to measure a household’s ability to access food within the last 30 days. Responses to these questions were recoded, summed and categorized as little to no hunger, moderate hunger, or severe hunger [36].

### ART adherence

ART Adherence was determined by asking participants: “Thinking over the past 7 days, have you missed any of your ARV doses or taken them at the wrong time?” Those who reported having missed or mistimed one or more doses in the past seven days were categorized as having recently missed or mistimed an ART dose.

### Socio-demographic

Socio-demographics explored included age, education, relationship status, parity, general health, asset-based socioeconomic status (SES), employment status, and bodily pain. Asset-based SES was constructed by asking participants about ownership of six assets: electricity, radio, television, landline telephone, computer, and refrigerator. Participants were also asked the type of fuel used for cooking (dichotomized into natural gas vs. other types) and water source (dichotomized into piped water vs. other sources). Polychoric correlation principal components analysis was used to construct asset-based SES quintiles [37, 38].

### Clinic characteristics

Ten study clinics in Yaoundé were included in the study. To balance clinic-level characteristics, constrained randomization was employed using four characteristics: clinic type, receipt of funding from PEPFAR, health district, and expected monthly number of WLWH seen at the clinic.

### Data analysis

Participant characteristics, and the prevalence of probable CMD, IPV, and household hunger were reported by ART adherence. Generalized estimating equations (GEE) modified log-Poisson regression models were used to explore the relationships between the three outcomes and ART adherence, in order to obtain risk ratio estimates [39]. We first examined these relationships in separate bivariate models to explore the unadjusted effects. Next, we fit multivariable models including all the primary exposures (probable CMD, IPV and household hunger) and adjusted for participant’s age, relationship status, bodily pain, and health district. Education was correlated with household hunger and was not included in multivariable models. General health was correlated with bodily pain and was not included in the multivariable models. All models accounted for clustering by study clinics using an exchangeable working correlation matrix, with a Kauermann-Carroll small sample correction applied to the standard errors using SAS PROC GLIMMIX [40].

Effect measure modification of the associations between the primary exposures and ART adherence by IPV was assessed by examining the statistical significance of an interaction term between each exposure separately and IPV, and the relative magnitudes of the model-estimated effects by IPV. A p-value of <0.20 guided interpretation of statistical significance of interaction terms [41]. Because the interaction term between IPV and household hunger reached statistical significance, multivariable analyses of the association between household hunger and ART adherence are presented both overall and stratified by IPV by estimating the effects from the models with the interaction term. All analyses were performed using SAS version 9.4 (SAS, Cary, NC).

## Results

The majority of study participants were 25 years or older (78%), started secondary school but did not finish (56%) and lived with a romantic partner (69%) (Table 1). Almost half (44%) of participants reported having missed or mistimed at least one ART dose in the past seven days. Approximately 42% of participants screened positive for probable CMD. Approximately one-third of participants reported physical or sexual IPV (36% and 31%, respectively). Fifteen percent reported moderate household hunger and 4% reported severe household hunger in the past 12 months (Table 2).

**Table 1:** Sociodemographic characteristics among pregnant women living with HIV in Yaoundé, Cameroon.

Variable	Total Sample	Recent missed/mistimed	No recent missed/mistimed
		ART dose	ART dose
	N (%)	N (%)	N (%)
**Total**	220 (100.0)	97 (44.1)	123 (55.9)
**Age**			
18–24	48 (21.8)	20 (20.6)	28 (22.8)
≥25	172 (78.2)	77 (79.4)	95 (77.2)
**Education**			
None to primary	42 (19.1)	15 (15.5)	27 (21.9)
Started secondary (not finish)	124 (56.4)	57 (58.8)	67 (54.5)
Completed secondary or more than secondary	54 (24.5)	25 (25.8)	29 (23.6)
**Relationship status: Living with romantic partner**			
No	69 (31.4)	32 (33.0)	37 (30.1)
Yes	151 (68.6)	65 (67.0)	86 (69.9)
**Parity**			
One pregnancy	21 (9.6)	10 (10.3)	11 (8.9)
2–3 pregnancies	94 (42.7)	35 (36.1)	59 (48.0)
4 or more pregnancies	105 (47.7)	52 (53.6)	53 (43.1)
**General health**			
Fair/Poor	62 (28.3)	39 (40.6)	23 (18.7)
Good	97 (44.3)	41 (42.7)	56 (45.5)
Very Good/ Excellent	60 (27.4)	16 (16.7)	44 (35.8)
Missing	1		
**Asset-based SES**			
Lowest Quintile	39 (17.7)	16 (16.5)	23 (18.7)
Lower Middle Quintile	40 (18.2)	20 (20.6)	20 (16.3)
Middle Quintile	53 (24.1)	23 (23.7)	30 (24.4)
Upper Middle Quintile	39 (17.7)	20 (20.6)	19 (15.4)
Upper Quintile	49 (22.3)	18 (18.6)	31 (25.2)
**Employment status**			
No	108 (49.5)	42 (43.3)	66 (54.6)
Yes	110 (50.5)	55 (56.7)	55 (45.4)
Missing	2		
**Bodily pain**			
None/ Very mild/ Mild	159 (72.3)	64 (66.0)	95 (77.2)
Moderate/ Severe	61 (27.7)	33 (34.0)	28 (22.8)
**Health district**			
Nkoldongo	139 (63.2)	49 (50.5)	90 (73.2)
Djoungolo	81 (36.8)	48 (49.5)	33 (26.8)

**Table 2:** Intimate partner violence, probable CMD, household hunger, and ART adherence among pregnant women living with HIV in Yaoundé, Cameroon.

Variable	Total Sample	Recent missed/mistimed	No recent missed/mistimed	Chi-Square p-value
		ART dose	ART dose	
	N (%)	N (%)	N (%)	
**Total**	220 (100.0)	97 (44.1)	123 (55.9)	
**Physical IPV**				0.82
No	138 (63.6)	59 (62.8)	79 (64.2)	
Yes	79 (36.4)	35 (37.2)	44 (35.8)	
Missing	3			
**Sexual IPV**				0.77
No	150 (69.1)	64 (68.1)	86 (69.9)	
Yes	67 (30.9)	30 (31.9)	37 (30.1)	
Missing	3			
**SRQ-20 score**				0.006
0–7	127 (57.7)	46 (47.4)	81 (65.9)	
≥8	93 (42.3)	51 (52.6)	42 (34.1)	
**Household hunger**				0.02
None/low	178 (80.9)	71 (73.2)	107 (87.0)	
Moderate	33 (15.0)	19 (19.6)	14 (11.4)	
Severe	9 (4.1)	7 (7.2)	2 (1.6)	

ART adherence was significantly associated with probable CMD. Approximately 53% of those who reported having missed or mistimed an ART dose screened positive for probable CMD compared to 34% of those who reported not having recently missed or mistimed an ART dose. Household hunger was also significantly associated with ART adherence. Approximately 27% of those who reported having recently missed or mistimed an ART dose reported moderate or severe household hunger compared to 13% of those who did not report a missed or mistimed ART dose. In bivariate analyses, neither physical nor sexual IPV was significantly associated with ART adherence.

As seen in Table 3, in multivariable analyses, probable CMD was associated with significantly greater risk of having recently missed or mistimed an ART dose (aRR = 1.39; 95% CI: 1.02, 1.91). Neither physical nor sexual IPV was associated with having missed or mistimed an ART dose in bivariate or multivariable models.

**Table 3:** Bivariate and multivariable associations between IPV, probable CMD, and ART adherence.

	Missed/mistimed ART dose	Missed/mistimed ART dose
	RR (95% CI)^a^	aRR (95% CI)^b^
**Physical or sexual IPV**		
No	1.00	1.00
Yes	1.05 (0.87, 1.26)	0.94 (0.75, 1.18)
**SRQ-20 score**		
0–7	1.00	1.00
≥8	1.49 (1.21, 1.83)	1.39 (1.02, 1.91)
**Household hunger**		
None/Low	1.00	1.00
Moderate/Severe	1.52 (1.01, 2.28)	1.33 (0.86, 2.08)
**Age**		
12–24		1.00
≥25		1.01 (0.76, 1.34)
**Living with romantic partner**		
Yes		1.00
No		0.93 (0.78, 1.11)
**Bodily pain**		
None/mild		1.00
Moderate/severe		1.27 (0.83, 1.93)
**Health district**		
Nkoldongo		1.00
Djoungolo		1.64 (1.09, 2.47)

^a^ Unadjusted and separate models

^b^ Model adjusted for age, living with romantic partner, bodily pain, and district

Interaction terms between household hunger and probable CMD and between IPV and probable CMD on ART adherence did not achieve statistical significance. Similarly, neither IPV nor household hunger appeared to modify the relationship between probable CMD and ART adherence when multivariable models were stratified first by IPV and then by household hunger.

**Fig 1:**
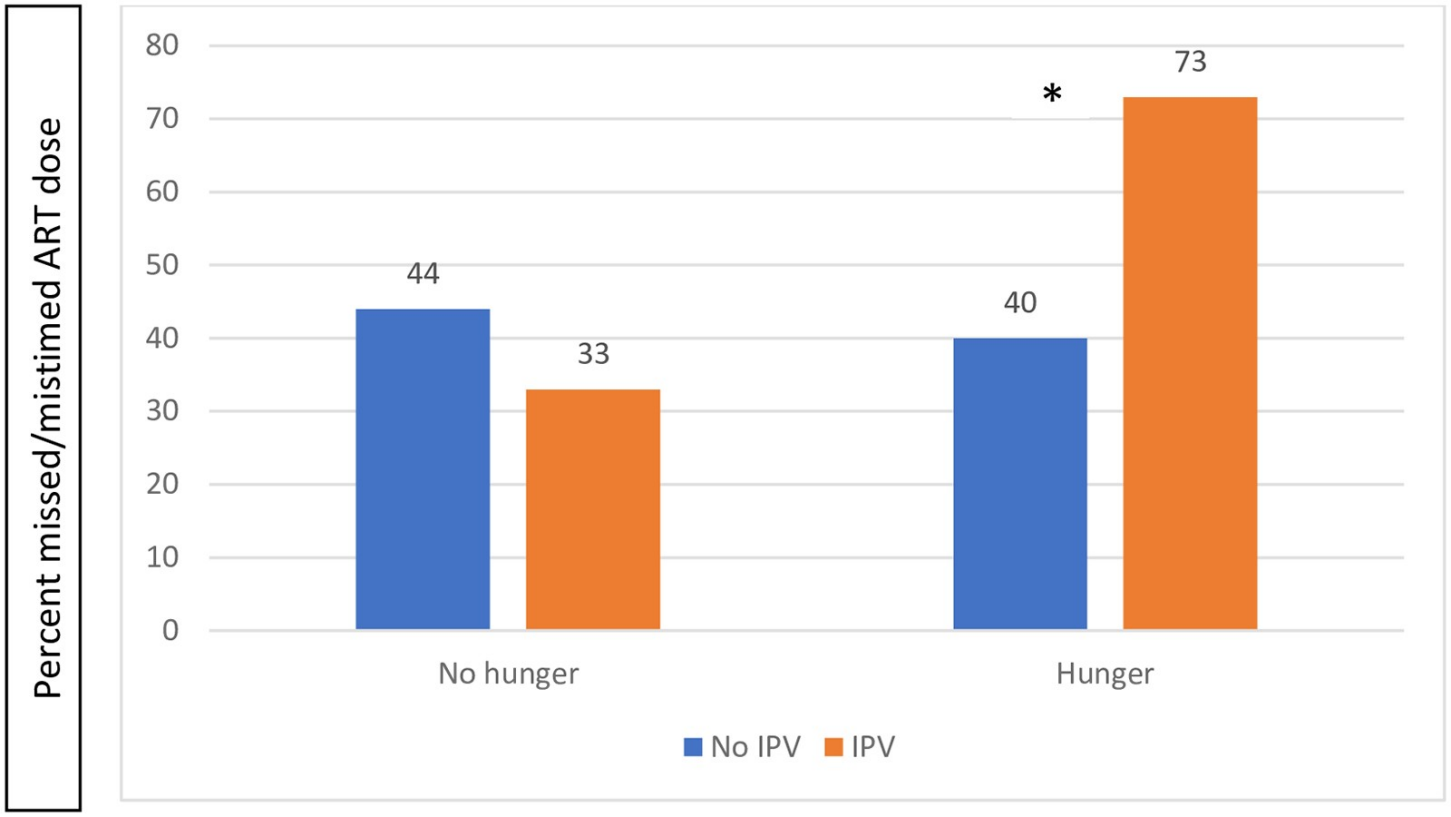
ART adherence and IPV among pregnant women living with HIV in Yaoundé, Cameroon stratified by household hunger. *denotes significance at p<0.05.

Effect measure modification by IPV appeared to be present when examining the relationship between household hunger and ART adherence. Among individuals who did not report IPV, there was no significant difference in the prevalence of having missed or mistimed an ART dose between those who reported moderate or severe levels of household hunger and those who reported no or low levels of household hunger (Fig 1). However, among those who reported IPV, the prevalence of having missed or mistimed an ART dose was significantly greater among those who reported moderate or severe household hunger compared to those who reported no or low levels of household hunger.

Similarly, the multivariable models provide evidence of modification of the association between hunger and ART adherence by IPV (multivariable interaction p-value = 0.146). Among those who experienced physical or sexual IPV, moderate or severe household hunger was significantly associated with greater risk of having missed or mistimed an ART dose (aRR 1.85 [95% CI: 1.22–2.79]) compared to those who reported no or low levels of household hunger. However, among those who did not report physical or sexual IPV, household hunger was not significantly associated with having missed or mistimed an ART dose (aRR = 0.78, 95% CI: 0.27–2.29) (Table 4).

**Table 4:** Bivariate and multivariable associations between household hunger and ART adherence stratified by IPV.

	Missed/mistimed ART dose			
	RR (95% CI)^a^	RR (95% CI)^b^	aRR (95% CI) ^a^^,^^c^	aRR (95% CI) ^b^^,^^c^
**Household hunger**				
No/low	1.0	1.0	1.0	1.0
Moderate/severe	0.90 (0.34, 2.40)	2.09 (1.43, 3.06)	0.78 (0.27, 2.29)	1.85 (1.22, 2.79)

^a^ Among those who report no physical or sexual IPV

^b^ Among those who report physical or sexual IPV

^c^ Model adjusted for age, living with romantic partner, bodily pain, and district

## Discussion

Suboptimal ART adherence was commonly reported among study participants, with almost half of study participants having recently missed or mistimed an ART dose. CMD and IPV were also commonly reported among this sample of pregnant WLWH in Cameroon. CMD was associated with suboptimal ART adherence. However, neither IPV nor household hunger modified the relationship between CMD and ART adherence. IPV modified the relationship between hunger and ART adherence. That is, hunger was associated with suboptimal ART adherence only among those who reported having experienced IPV.

Previous estimates of suboptimal ART adherence among pregnant WLWH in Cameroon were lower than reported in this study. A study of pregnant or breastfeeding WLWH in Cameroon found that 20% of study participants reported moderate ART adherence and 7% reported low ART adherence (measured using a four-item self-report questionnaire) six months after initiating Option B+ [42]. A study of PLWH (men and women) in Cameroon found that between 23% and 35% of participants reported recent non-adherence to ART regimens [8]. Estimates of suboptimal ART adherence among pregnant WLWH across other sub-Saharan African countries vary but are also generally lower than reported in this study [43]. Studies of pregnant WLWH in Kenya, Nigeria, Zimbabwe, and South Africa reported levels of ART non-adherence ranging from 16% to 31% [44–47]. Caution should be taken when comparing results across studies due to differences in the populations surveyed and methods used to measure ART adherence.

Despite the implementation and scale-up of Option B+, evidence-based interventions to improve ART adherence among pregnant WLWH are urgently needed. Such interventions may need to be developed or adapted to enhance the acceptability and effectiveness for pregnant WLWH, as previous studies of interventions to improve ART adherence among pregnant WLWH in sub-Saharan Africa have largely found limited effectiveness [48–50]. Additional research is needed to identify effective and culturally appropriate strategies to promote sustained ART adherence among pregnant WLWH throughout sub-Saharan Africa.

Probable CMD was commonly reported and associated with suboptimal ART adherence which aligns with existing literature [10, 51–54]. Similar to many resource-constrained settings, the availability of evidence-based mental health care is limited throughout Cameroon. There is an urgent need to expand and strengthen the mental health workforce throughout Cameroon [55]. The WHO Mental Health Gap Action Programme (mhGAP) provides guidance for integrating mental health care into non-specialized services in resource-constrained settings [56]. The feasibility and acceptability of implementing the mhGAP guidance into PMTCT services in Cameroon should be explored. Evidence-based screening and referral to care for CMD, particularly depression, should be integrated into PMTCT services, as screening and treatment of depression has been associated with improved ART adherence among PLWH [57]. Less is known about the impact of the treatment of anxiety on ART adherence among PLWH. The extent to which mental health treatment improves ART adherence among pregnant WLWH remains unknown. A pilot study of an intervention that combined peer mentoring and cognitive-behavioral components was associated with reduced depression scores and better attendance at medical visits among pregnant WLWH in South Africa [58]. However, findings related to ART adherence were not reported. A randomized control trial of an interactive group counseling intervention for pregnant WLWH was not significantly associated with depression post-intervention [59]. Additional research to understand how to manage CMD and promote ART adherence among pregnant WLWH is needed. Development and evaluation of integrated interventions that address CMD and ART adherence among pregnant WLWH is warranted.

Household hunger and IPV were commonly reported among pregnant WLWH. Effect measure modification appeared to be present between IPV and hunger. Household hunger appeared to be associated with suboptimal ART adherence only among those who reported IPV. Previous research has found food insecurity and IPV to be independently associated with suboptimal ART adherence among pregnant WLWH [60]. The current work is among the first to examine effect modification of the relationship between hunger and ART adherence by IPV among pregnant WLWH. This is significant as pregnant WLWH who experience IPV and household hunger appear to be particularly vulnerable to suboptimal ART adherence. Multidimensional interventions that address food insecurity, IPV, and ART adherence are needed and should be integrated into PMTCT service settings. A syndemic understanding of the interrelationships between IPV, hunger, and ART adherence among pregnant WLWH can inform the adaptation and implementation of interventions to improve ART adherence among this population and may contribute to improved maternal and child health outcomes. Interventions focused on food assistance and livelihood interventions have shown promise in improving food security and HIV care outcomes among PLWH [61]. More research is needed to understand the effectiveness of these interventions among pregnant WLWH. Strategies to adapt such interventions for women experiencing IPV or to incorporate IPV programming into food or livelihood interventions may yield promising insights. The effectiveness of integrated interventions on addressing food insecurity, IPV, and mental health disorders should be evaluated.

The mechanisms through which IPV and household hunger interact to exacerbate suboptimal ART adherence warrant investigation. Further research should investigate mechanisms linking food insecurity and ART adherence among pregnant WLWH and why this relationship is exacerbated in the presence of IPV. Longitudinal research may be particularly suited to advancing our understanding of the relationship between food insecurity, IPV, and ART adherence among pregnant WLWH.

Limitations of the study should be noted. Data collection was restricted to Yaoundé, limiting the representativeness of the data. Findings of this study may not be generalizable to other regions in Cameroon. Due to the cross-sectional study design, causality and the temporal associations described in this study cannot be inferred. Further, the measures of probable CMD, IPV, household hunger, and ART adherence relied on self-reported data. Further, our syndemic analyses did not explore the role of substance use, a core component of the original SAVA syndemic. Future research should examine to what extent substance use influences relationships among IPV, hunger, mental health, and ART adherence among pregnant WLWH and other vulnerable populations. Finally, and most importantly, given the relatively small sample size used in this analysis, caution should be used in interpreting these findings.

A syndemic approach to programming in support of optimal ART adherence for pregnant WLWH in Cameroon is critical. HIV services for pregnant WLWH should directly address mental health, food insecurity and IPV in tandem to achieve ART adherence goals. Routine screening for CMD, IPV and food insecurity during ANC services with robust referral systems for comprehensive services can complement adherence counselling during PMTCT and antenatal visits.

## Supporting information

S1 File (PDF)
