# Supplementary material for: The syndemic effects of mental ill health, household hunger, and intimate partner violence on adherence to antiretroviral therapy among pregnant women living with HIV in Yaoundé, Cameroon
Source: PLoS One. 2021 Feb 19;16(2):e0246467. doi: 10.1371/journal.pone.0246467 (PMC7894814; doi:10.1371/journal.pone.0246467)
Supplement: S1 File — (PDF) [file pone.0246467.s001.pdf]

PRENATAL SURVEY (1st Survey)	
**Clinic ID:** |____|____|	**Participant ID:** |____|____|____|
**Observation Date:** |____|____| / |____|____| / |____|____|____|____| *DD MM YYYY*	**RA ID:** |____|____|

Les informations contenues dans ce questionnaire sont confidentielles, conformément à la loi N° 91/023 du 16 décembre 1991 portant sur les enquêtes statistiques au Cameroun.

INTERVIEWER NOTE : TO BE COMPLETED SAME DAY AS SCREENING & CONSENT

SECTION 0: CHECKLIST

**S0Q01**	Screened eligible *0. No 1. Yes*	|___|
**S0Q02**	Consent signed *0. No 1. Yes*	|___|
**S0Q03**	Contact section completed *0. No 1. Yes*	|___|
**S0Q04**	Survey completed *0. No 1. Yes*	|___|
**S0Q05**	Correct hospital / clinic is selected at the top of first page of data collection 0. No 1. Yes	|___|
**S0Q06**	Participant ID is written at the top of each page 0. No 1. Yes	|___|
**S0Q07**	Date is written at the top of each page 0. No 1. Yes	|___|
**S0Q08**	Double-check that all information has been completed before ending the visit *0. No 1. Yes*	|___|
**S0Q09**	Remind mother of next visit date *(approximate)* *0. No 1. Yes*	|___|
**S0Q10**	Incentive was given out and incentive paperwork was completed *0. No 1. Yes*	|___|
**S0Q11**	Medical extraction completed *0. No 1. Yes*	|___|

PRENATAL SURVEY (1st Survey)	
Clinic ID: |____|____|	Participant ID: |____|____|____|
Observation Date: |____|____| / |____|____| / |____|____|____|____| DD MM YYYY	RA ID: |____|____|

Thank you for joining Healthy Baby – Cameroon. Today is the first of four interviews. On each visit, we'll talk through similar survey questions. On our next visits, once your baby is born, we'll also ask questions from a short questionnaire called "Ages and Stages" that asks about your baby and if you have concerns.

Do you have any questions before we begin?

First, I'd like to start by getting some of your contact information.

SECTION 1: CONTACT INFORMATION

**S1Q01**	What is the best phone number we can use to call you (to schedule appointment, to send a reminder about an appointment)?<br>*[Enter number and repeat back to respondent to confirm if correct.]*<br>________________________________	
**S1Q02**	May we call this number?<br>*00. No 01. Yes*	|___||___|
**S1Q03**	May we text you at this number?<br>*00. No 01. Yes*	|___||___|
**S1Q04**	Which do you prefer, a call or a text?<br>*01. Call 02. Text 03. No preference*	|___||___|
**S1Q05**	When I call and/or text, may I mention that you are part of Healthy Baby – Camerooon?<br>*00. No 01. Yes*	|___||___|
**S1Q06**	Do you have a second phone number we can use to call you (to schedule appointment, to send a reminder about an appointment)?<br>*[Enter number and repeat back to respondent to confirm if correct.]*<br>________________________________	
**S1Q07**	May we call this number?<br>*00. No 01. Yes*	|___||___|
**S1Q08**	May we text you at this number?<br>*00. No 01. Yes*	|___||___|
**S1Q09**	Which do you prefer, a call or a text?<br>*01. Call 02. Text 03. No preference*	|___||___|
**S1Q10**	When I call and/or text, may I mention that you are part of Healthy Baby – Camerooon?<br>*00. No 01. Yes*	|___||___|

PRENATAL SURVEY (1st Survey)	
**Clinic ID: |____|____|**	**Participant ID: |____|____|____|**
**Observation Date: |____|____| / |____|____| / |____|____|____|____|** DD MM YYYY	**RA ID: |____|____|**

If we are unable to reach you at those numbers (for example, if your phone number changes), who can we call, to try to reach you?

**S1Q11**	*Additional Name 1: [Record]* ______________________________	
**S1Q12**	*Relation : [Record]* ______________________________	
**S1Q13**	*Phone number(s) Record]* ______________________________	
**S1Q14**	*Is it okay to say that you are participating in Healthy Baby - Cameroon to this person?* *0. No 1. Yes*	|___|
**S1Q15**	*Additional Name 2:* ______________________________	
**S1Q16**	*Relation : [Record]* ______________________________	
**S1Q17**	*Phone number(s) Record]* ______________________________	
**S1Q18**	*Is it okay to say that you are participating in Healthy Baby - Cameroon to this person?* *0. No 1. Yes*	|___|
**S1Q19**	*Additional Name 3:* ______________________________	
**S1Q20**	*Relation : [Record]* ______________________________	
**S1Q21**	*Phone number(s) Record]* ______________________________	
**S1Q22**	*Is it okay to say that you are participating in Healthy Baby - Cameroon to this person?* *0. No 1. Yes*	|___|

SECTION 2. HOSPITAL / CLINIC

**0_S201**	Which clinic or hospital does the participant attend?<br>01 =<br>02 =<br>03 = *REDACTED CLINIC NAMES*<br>04 =<br>05 =<br>06 =<br>07 =<br>08 =<br>09 =<br>10 =	|___||___|

SECTION 3. MATERNAL DEMOGRAPHICS**Thank you for that contact information. I would now like to ask you some brief questions about yourself.**

For all questions : 88= Don't Know/Not applicable ; 99=Missing/Refused

**0_DE01**	How old were you at your last birthday? *[If unknown, enter best guess.]*	|___||___|
**0_DE02**	What is your relationship status now? Are you:<br>01 = Married and living with your husband<br>02 = Married and not living with your husband<br>03 = Living with a romantic partner whom you are not married to<br>04 = With romantic partner but not married to nor living together<br>05 = Widowed<br>06 = Divorced<br>07 = Separated, or<br>08 = single?	|___||___|
**0_DE03**	What is your religion or belief system?<br>01 = Catholic<br>02 = Protestant<br>03 = Muslim<br>04 = Animiste<br>05 = Eglise de reveil (Evangelical)<br>06 = None<br>10 = Autres (*specify*) : ____	|___||___|
**0_DE04**	What is the highest level of education you have completed?<br>01 = No Education<br>02 = Started Primary School, Did Not Finish<br>03 = Completed Primary<br>04 = Started Secondary School, Did Not Complete<br>05 = Completed Secondary School<br>06 = More than Secondary School ("Supereur")	|___||___|
**0_DE05**	As you know, some people take up jobs for which they are paid in cash or in-kind. By "in-kind" I mean are you paid in food or other goods or services. Others sell things, have a small business or work on the family farm or in the family business. We have a couple of questions about any work you do for cash or in-kind services.<br>Have you worked in any capacity in the past 3 months?<br>*00. No 01. Yes*	|___||___|
**0_DE06**	Were you paid in cash or in-kind for this work?<br>01 = Cash only<br>02 = Cash and in-kind<br>03 = In-kind only<br>04 = Not paid	|___||___|

Clinic ID: |____|____| **Participant ID:** |____|____|____|

Observation Date: |____|____| / |____|____| / |____|____|____|____| **RA ID:** |____|____|
DD MM YYYY

SECTION 4. MATERNAL HEALTH: PREGNANCY HISTORY, CHILDREN, AND CURRENTLY BREASTFEEDING

Next I would like to better understand your health history, in terms of pregnancies.

[Interviewer fills out grid below, with mother's answers to these questions]

**0_PG01**	How many times have you become pregnant, including this one?<br>*[For 2, enter 02, etc…] Skip to S4Q## if this is the woman's first pregnancy.*	|___||___|

Pregnancy #	**0_PG02A-J**<br>**What was the outcome of each pregnancy? Miscarriage, stillborn, termination, live birth?**<br>*01 = Born alive*<br>*02 = Not Born alive*	**0_PG03A-J**<br>**Age of child**<br>*77. If born alive and has since died*	**0_PG04A-J**<br>**Breastfeeding now?**<br>*[If living]*<br>*00 = No*<br>*01 = Yes*
1st	|___||___|	|___||___|	|___||___|
2nd	|___||___|	|___||___|	|___||___|
3rd	|___||___|	|___||___|	|___||___|
4th	|___||___|	|___||___|	|___||___|
5th	|___||___|	|___||___|	|___||___|
6th	|___||___|	|___||___|	|___||___|
7th	|___||___|	|___||___|	|___||___|
8th	|___||___|	|___||___|	|___||___|
9th	|___||___|	|___||___|	|___||___|
10th	|___||___|	|___||___|	|___||___|

**0_PG05**	Have you had any children who were born alive but have since died?<br>*[If participant already mentioned death of child, do not ask again, but record yes.]*<br>*00. No 01. Yes*	|___||___|

Now I would like to ask you about how you communicated and interacted with [*OLDEST, 2nd CHILD, 3rd CHILD, …, YOUNGEST*] during your pregnancy with him/her.

When you were pregnant with [*YOUR OLDEST, etc.*], how often did you do the following?
[READ OPTIONS ALOUD.]

00 = Never
01 = Rarely
02 = Sometimes
03 = Frequently

	**0_PG06** Talk softly to him/her and gently touch your belly?	**0_PG07** Sing songs to him/her?	**0_PG08** Tell him/her about his/her family?	**0_PG09** Dance to music or the radio?	**0_PG10** Encourage your older children to touch your belly & talk to him/her?	**0_PG11** Encourage partner/husb to talk & touch your belly?
	|___||___|	|___||___|	|___||___|	|___||___|	|___||___|	|___||___|

With your current pregnancy, how often did you do the following?

	**0_PG12** Talk softly to him/her & touch belly?	**0_PG13** Sing songs to him/her?	**0_PG14** Tell him/her about his/her family?	**0_PG15** Dance to music or the radio?	**0_PG16** Encourage older children to touch & talk?	**0_PG17** Encourage partner/husb to talk & touch?
	|___||___|	|___||___|	|___||___|	|___||___|	|___||___|	|___||___|

PRENATAL SURVEY (1st Survey)	
Clinic ID: |____|____|	Participant ID: |____|____|____|
Observation Date: |____|____| / |____|____| / |____|____|____|____| DD MM YYYY	RA ID: |____|____|

Thank you. The next questions ask for your views about your health, such as how you have been feeling and how well you are able to do your usual activities.

For each of the following questions, please select the response that best describes how you have been feeling. If you are unsure about how to answer a question, please give the best answer you can.

Short Form – 20 Health Survey		
**0_SF01**	In general, would you say your health is:<br>*[READ OPTIONS ALOUD]*<br>01 = Excellent<br>02 = Very Good<br>03 = Good<br>04 = Fair<br>05 = Poor	|___||___|
	**For how long (if at all) has your health limited you in each of the following activities?**<br>*[FOR S4QX – S4QXX, READ OPTIONS ALOUD WITH EACH QUESTION]*<br>00 = Not limited at all<br>01 = Limited for 3 months or less<br>02 = Limited for more than 3 months	
**0_SF02**	The kinds or amounts of vigorous activities you can do, like lifting heavy objects, running or participating in strenuous sports	|___||___|
**0_SF03**	The kinds or amounts of moderate activities you can do, like moving a table, carrying groceries, or going to the fields, carrying water	|___||___|
**0_SF04**	Walking uphill or climbing a few flights of stairs	|___||___|
**0_SF05**	Bending, lifting, or stooping	|___||___|
**0_SF06**	Walking one block	|___||___|
**0_SF07**	Eating, dressing, bathing, or using the toilet	|___||___|
**0_SF08**	How much bodily pain have you had in the **past 4 weeks**?<br>*[READ OPTIONS ALOUD]*<br>00 = None<br>01 = Very Mild<br>02 = Mild<br>03 = Moderate<br>04 = Severe<br>05 = Severe	|___||___|
**0__SF09**	During the **past 4 weeks**, how much energy (la force) did you have?<br>*[READ OPTIONS ALOUD]*<br>00 = None	|___||___|

	01 = A little<br>02 = Some<br>03 = Quite a lot<br>04 = Very Much	
**0_SF10**	Does your health keep you from working at a job, doing work around the house, or going to school?<br>*[READ OPTIONS ALOUD]*<br>00 = No<br>01 = Yes, for 3 months or less<br>02 = Yes, for more than 3 months	|___||___|
**0_SF11**	Have you been unable to do certain kinds or amounts of work, housework, or schoolwork because of your health?<br>[READ *OPTIONS ALOUD]*<br>00 = No<br>01 = Yes, for 3 months or less<br>02 = Yes, for more than 3 months	|___||___|
	**For each of the following questions, please choose the answer that comes closest to the way you have been feeling during the past month.**<br>*[FOR S4QX – S4QXX, READ OPTIONS ALOUD WITH EACH QUESTION]*<br>00 = None of the time<br>01 = A little of the time<br>02 = Some of the time<br>03 = A good bit of the time<br>04 = Most of the time<br>05 = All of the time	
**0_SF12**	How much of the time, during the past month, have you been a very nervous person?	|___||___|
**0_SF13**	During the past month, how much of the time have you felt calm and peaceful?	|___||___|
**0_SF14**	How much of the time, during the past month, have you felt downhearted and blue?	|___||___|
**0_SF15**	During the past month, how much of the time have you been a happy person?	|___||___|
**0_SF16**	How often, during the past month, have you felt so down in the dumps that nothing could cheer you up?	|___||___|
	**For the following questions (S4QX-QX) please choose the answer that best describes whether each of the following statements is true or false for you.**<br>*[FOR S4QX – S4QXX, READ OPTIONS ALOUD WITH EACH QUESTION]*<br>01 = Definitely true<br>02 = Mostly true<br>03 = Not sure<br>04 = Mostly false	

	05 = Definitely false	
**0_SF17**	I am somewhat ill.	|___||___|
**0_SF18**	I am as healthy as anybody I know.	|___||___|
**0_SF19**	My health is excellent.	|___||___|
**0_SF20**	I have been feeling bad lately	|___||___|
**0_SF21**	How long does it take you to walk to the nearest clinic or health care center, where you would normally go for standard (not emergency) health needs? *[Enter number of minutes. Record a "0" or "00" in front of the minutes if necessary. E.g., 15 minutes should be entered as 015.]*	|___|___|___|
**Household Hunger Scale**		
**0_HG01**	How often did this happen in the past [4 weeks/30 days]?<br>01 = Rarely (1-2 times)<br>02 = Sometimes (3-10 times)<br>03 = Not sure (More than 10 times)	|___|___|
**0_HG02**	In the past [4 weeks/30 days], did you or any household member go to sleep at night hungry because there was not enough food?<br>00 = No → **SKIP TO S4Q42**<br>01 = Yes<br>88/99 = DK/Refused → **SKIP TO S4Q42**	|___||___|
**0_HG03**	How often did this happen in the past [4 weeks/30 days]?<br>01 = Rarely (1-2 times)<br>02 = Sometimes (3-10 times)<br>03 = Not sure (More than 10 times)	|___||___|
**0_HG04**	In the past [4 weeks/30 days], did you or any household member go a whole day and night without eating anything at all because there was not enough food?<br>00 = No → **SKIP TO S4Q44**<br>01 = Yes<br>88/99 = DK/Refused → **SKIP TO S4Q44**	|___||___|
**0_HG05**	How often did this happen in the past [4 weeks/30 days]?<br>01 = Rarely (1-2 times)<br>02 = Sometimes (3-10 times)<br>03 = Not sure (More than 10 times)	|___||___|
**0_HG06**	In the past 4 weeks (30 days), did you or any household member have to eat some foods that you really did not want to eat because of lack of resources to obtain other types of food?<br>00 = No → **SKIP TO S4Q46**	|___||___|

	01 = Yes<br>88/99 = DK/Refused → **SKIP TO S4Q46**	
**0_HG07**	How often did this happen in the past [4 weeks/30 days?<br>01 = Rarely (1-2 times)<br>02 = Sometimes (3-10 times)<br>03 = Not sure (More than 10 times)	|___||___|
**0_HG08**	In the past 4 weeks (30 days), did you or any household member have to eat a smaller meal than you felt you needed because there was not enough food?<br>00 = No → **SKIP TO S4Q48**<br>01 = Yes<br>88/99 = DK/Refused → **SKIP TO S4Q48**	|___||___|
**0_HG09**	How often did this happen in the past [4 weeks/30 days]?<br>01 = Rarely (1-2 times)<br>02 = Sometimes (3-10 times)<br>03 = Not sure (More than 10 times)	|___||___|
**0_HG10**	In the past 4 weeks (30 days), did you or any household member have to eat fewer meals in a day because there was not enough food?<br>00 = No → **SKIP TO S4Q50**<br>01 = Yes<br>88/99 = DK/Refused → **SKIP TO S4Q50**	|___||___|
**0_HG11**	How often did this happen in the past [4 weeks/30 days]?<br>01 = Rarely (1-2 times)<br>02 = Sometimes (3-10 times)<br>03 = Not sure (More than 10 times)	|___||___|
**0_HG12**	In the past 4 weeks (30 days), was there ever no food to eat of any kind in your house because of lack of resources to get food?<br>00 = No → **SKIP TO S4Q52**<br>01 = Yes<br>88/99 = DK/Refused → **SKIP TO S4Q52**	|___||___|
**0_HG13**	How often did this happen in the past [4 weeks/30 days]?<br>01 = Rarely (1-2 times)<br>02 = Sometimes (3-10 times)<br>03 = Not sure (More than 10 times)	|___||___|
**0_HG14**	In the past 4 weeks (30 days), did you or any household member go to sleep at night hungry because there was not enough food?<br>00 = No → **SKIP TO S4Q54**<br>01 = Yes	|___||___|

	88/99 = DK/Refused → **SKIP TO S4Q54**	
**0_HG15**	How often did this happen in the past [4 weeks/30 days]?<br>01 = Rarely (1-2 times)<br>02 = Sometimes (3-10 times)<br>03 = Not sure (More than 10 times)	|___||___|
**0_HG16**	In the past 4 weeks (30 days), did you or any household member go a whole day and night without eating anything because there was not enough food?<br>00 = No → **SKIP TO S4Q56**<br>01 = Yes<br>88/99 = DK/Refused → **SKIP TO S4Q56**	|___||___|
**0_HG17**	How often did this happen in the past [4 weeks/30 days]?<br>01 = Rarely (1-2 times)<br>02 = Sometimes (3-10 times)<br>03 = Not sure (More than 10 times)	|___||___|
**Anti-retroviral Adherence**		
**0_ARV01**	When you feel better, do you sometimes stop taking your medicine?<br>*00. No 01. Yes*	|___||___|
**0_ARV02**	Thinking over the past 7 days, have you missed any of your ARV doses or taken them at the wrong time?<br>00 = No → **SKIP TO S4Q59**<br>01 = Yes<br>88/99 = DK/Refused → **SKIP TO S4Q59**	|___||___|
**0_ARV03**	In the past 7 days, how often have you missed dose of your ARVs (anti-retroviral medicines)? *[Enter number of doses missed.]*	|___||___|
**0_ARV04**	Sometimes if you feel worse when you take the medicine, do you stop talking it?<br>*00. No 01. Yes*	|___||___|
**0_ARV05**	***Ask the mother to think back over the past seven days and identify the times when she missed a dose.***<br><br>**Show the mother a copy of this visual analogue scale** (BELOW), or an unmarked enlarged version. While placing your finger on the appropriate place, tell the mother that if she had taken all medicine doses to point to 10. If the mother missed all the doses, she would point to 0—in the meantime, you move your finger to 0.<br><br>**Now give the mother an opportunity to point out her level of adherence.** The interviewer then marks the visual analogue scale. *[Record the whole number where the mother pointed.]*	|___||___|

	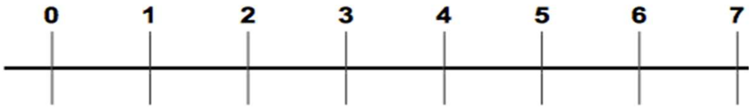	
**0_ARV06**	***Ask the mother to think back over the past seven days and identify the times when she took it at the wrong time.***<br><br>**Now give the mother an opportunity to point out her level of adherence.** The interviewer then marks the visual analogue scale. *[Record the whole number where the mother pointed.]*<br>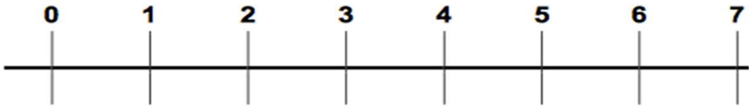	|___||___|
**Self-Reporting Questionnaire (SRQ-20)**		
**Thank you! Now I'd like you to think about the past 30 days and how you have felt during that time.**<br>00 = No<br>01 = Yes		
**0_SRQ01**	Do you often have headaches?	|___||___|
**0_SRQ02**	Is your appetite poor?	|___||___|
**0_SRQ03**	Do you sleep badly?	|___||___|
**0_SRQ04**	Are you easily frightened?	|___||___|
**0_SRQ05**	Do your hands shake?	|___||___|
**0_SRQ06**	Do you feel nervous, tense or worried?	|___||___|
**0_SRQ07**	Is your digestion poor?	|___||___|
**0_SRQ08**	Do you have trouble thinking clearly?	|___||___|
**0_SRQ09**	Do you feel unhappy?	|___||___|

0_SRQ10	Do you cry more than usual?	|___||___|
0_SRQ11	Do you find it difficult to enjoy your daily activities?	|___||___|
0_SRQ12	Do you find it difficult to make a decision?	|___||___|
0_SRQ13	Is your daily work suffering?	|___||___|
0_SRQ14	Are you unable to play a useful part in life?	|___||___|
0_SRQ15	Have you lost interest in things?	|___||___|
0_SRQ16	Do you feel that you are a worthless person?	|___||___|
0_SRQ17	Has the thought of ending your life been on your mind?	|___||___|
0_SRQ18	Do you feel tired all the time?	|___||___|
0_SRQ19	Do you have uncomfortable feelings in your stomach?	|___||___|
0_SRQ20	Are you easily tired?	|___||___|
	**Intimate Partner Violence**	
0_DV01	*Check for presence of others: Do not continue until effective privacy is ensured.*<br>00 = Privacy not possible → **SKIP to Section 5 (Household)**<br>01 = Privacy obtained	|___||___|
	**Now I would like to ask you questions about some other important aspects of a woman's life. I know that some of these questions are very personal. Your answers are helpful in understanding your experiences. If you are uncomfortable or prefer not to answer, you do not have to answer these questions. Let me assure you that your answers are completely confidential and will not be told to anyone without your permission.**<br>*[These questions concern all women regardless of their relationship status.]*	
	**When two people marry or live together, they share both good and bad moments. In your relationship with your (last) husband/partner do (did) the following happen frequently, only sometimes, or never?**<br>01 = Frequently<br>02 = Sometimes<br>03 = Never	
0_DV02	He usually spends/spent his free time with you?	|___||___|
0_DV03	He (consults/consulted) you on different household matters?	|___||___|

0_DV04	He (is/was) affectionate with you ?	|___||___|
0_DV05	He (respects/respected) you and your wishes ?	|___||___|
	**Now I am going to ask you about some situations which happen to some women. Please tell me if these apply to your relationship with your (last) husband/partner**?<br>00 = No<br>01 = Yes<br>88 = Don't Know	
0_DV06	He (is/was) jealous or angry if you (talk/talked) to other men?	|___||___|
0_DV07	He frequently (accuses/accused) you of being unfaithful?	|___||___|
0_DV08	He (does/did) not permit you to meet your girl friends	|___||___|
0_DV09	He (tries/tried) to limit your contact with your family	|___||___|
0_DV10	He (insists/insisted) on knowing where you (are/were) at all time?	|___||___|
0_DV11	He (does/did) not trust you with any money?	|___||___|
	**Now if you will permit me, I need to ask some more questions about your relationship with your (last) husband/partner. In the last 12 months, has your (last) husband/partner:**<br>00 = No<br>01 = Yes<br>88 = Don't Know	
0_DV12	Said or done something to humiliate you in front of others ?	
0_DV13	Threatened you or someone close to you with harm?	|___||___|
0_DV14	Insulted or belittled you?	|___||___|
0_DV15	Pushed you, shaken you, or thrown something at you?	|___||___|
0_DV16	Slapped you?	|___||___|
0_DV17	Twisted your arm, or pulled your hair?	|___||___|
0_DV18	Punched you with his fist or with something that could hurt you ?	|___||___|
0_DV19	Kicked or dragged you?	|___||___|
0_DV20	Tried to strangle you or burn you ?	|___||___|
0_DV21	Threatened you with a knife, a gun, or other type of weapon ?	|___||___|
0_DV22	Attacked you with a knife, a gun, or other type of weapon ?	|___||___|

0_DV23	Physically forced you to have sexual intercourse with him even when you did not want to?	|___||___|
0_DV24	Forced you to perform other sexual acts you did not want to?	|___||___|
**Section 5. Household Questions**		
0_HH01	In total, how many people are usually living in your home (adults and children)?	|___||___|
0_HH02	Of those in your household, how many are older than 18 years of age?	|___||___|
0_HH03	How many of those older than 18 in your household are female?	|___||___|
0_HH04	Male?<br>*[Check that the total of males and females over 18 matches earlier total. If not, ask for clarification.]*	|___||___|
0_HH05	Of those in your household, how many are under the age of 18?	|___||___|
0_HH06	How many of those under 18 in your household are female?	|___||___|
0_HH07	Male?<br>*[Check that the total of males and females under 18 matches earlier total. If not, ask for clarification.]*	|___||___|
0_HH08	How many children younger than 18 attend school?	|___||___|
0_HH09	What is the main source of drinking water for members of your household?<br><br>**PIPED WATER**<br>11 = Piped into dwelling<br>12 = Piped to yard/plot<br>13 = Piped to neighbor<br>14 = Public tap/Standpipe<br><br>21 = TUBE WELL OR BOREHOLE<br><br>**DUG WELL**<br>31 = Protected well<br>32 = Unprotected well<br><br>**WATER FROM SPRING**<br>41 = Protected spring<br>42 = Unprotected spring<br><br>51 = Rainwater<br>61 = Tanker truck<br>71 = Surface water (river, da, lake, pond, etc.)	|___||___|

	81 = Bottled water<br>96 = Other (specify):<br>___________________________________	
	Does your household have :<br>*00 = No*<br>*01 = Yes*	
**0_HH10**	Electricity ?	|___||___|
**0_HH11**	A radio ?	|___||___|
**0_HH12**	A television ?	|___||___|
**0_HH13**	A non-mobile telephone ?	|___||___|
**0_HH14**	A computer ?	|___||___|
**0_HH15**	A refrigerator ?	
**0_HH16**	What type of fuel does your household mainly use for cooking?<br>01 = Electricity<br>03 = Natural Gas<br>04 = Biogas/Kerosene<br>07 = Charcoal/Coal<br>08 = Wood<br>09 = Straw/Shrubs/Grass<br>10 = Agricultural crop<br>11 = Animal dung<br>95 = No food cooked in household<br>96 = Other (specify):<br>___________________________________	|___||___|
	**CLOSING**	

Thank you much for your time. We appreciate your help. Do you have any questions about what we have discussed or what happens to the information?

Our next meeting will be on at your baby's 6-month check-up, which will probably be during [*ESTIMATE MONTH BASED ON DATE OF BIRTH*]. If anything changes, will you let me know? Here is my phone number – [*PROVIDE PHONE NUMBER*]. I look forward to seeing you then!

Give incentive and complete form with maternal signature to confirm receipt of incentive.

MEDICAL INFORMATION

(Medical abstraction, 0_ME01, 0_ME02,…)
